# Supplementary material for: Studies Using Mutant Strains of Azospirillum brasilense Reveal That Atmospheric Nitrogen Fixation and Auxin Production Are Light Dependent Processes
Source: Microorganisms. 2023 Jun 30;11(7):1727. doi: 10.3390/microorganisms11071727 (PMC10383956; doi:10.3390/microorganisms11071727)
Supplement: Supplementary file 1 [file microorganisms-11-01727-s001.zip › Ferrieri_Microorganisms 2459261 Supplemental Materials.pdf]

microorganisms

MDPI

Supplemental Material

Biological Functions of *Azospirillum brasilense* that can Benefit Host Growth are Responsive to Light Stimuli.

Alexandra Bauer Housh[1,2 3], Randi Noel[1,3,4], Avery Powell[1,3,5], Spenser Waller[1,3,5], Stacy L. Wilder[1], Stephanie Sopko[1,6,†], Mary Benoit[1,4], Garren Powell[1,6], Michael J. Schueller[1,2], Richard A. Ferrieri[1,2,3,4,*]

[1.] Missouri Research Reactor Center, University of Missouri, Columbia, MO 65211 (A.B.H., alibauer1109@gmail.com; R.N., rlnhmh@umsystem.edu; A.P., avery.snyder00@gmail.com; S.L.W., wildersl@missouri.edu; S.S., sa207945@atsu.edu; M.B., mvbf4w@mail.missouri.edu; G.P., gmp722@health.missouri.edu; S.W., spenserwaller@gmail.com; M.J.S., schuellerm@missouri.edu; R.A.F., ferrierir@missouri.edu),
[2.] Chemistry Department, University of Missouri, Columbia, MO 65211,
[3.] Interdisciplinary Plant Group, University of Missouri, Columbia, MO 65211,
[4.] Division of Plant Science & Technology, University of Missouri, Columbia, MO 65211,
[5.] School of Natural Resources, University of Missouri, Columbia, MO 65211,
[6.] Department of Biochemistry, University of Missouri, Columbia, MO 65211,
† *Present Address*: Osteopathic School of Medicine, A.T. Still University of Health Sciences, Kirksville, MO 63501, USA.

* *Correspondence*: Richard A. Ferrieri (Email: ferrierir@missouri.edu, Tel: 1-573-882-5211)

Citation: Housh, A.B.; Noel, R.; Powell, A.; Waller, S.; Wilder, S.L.; Sopko, S.; Benoit, M.; Powell, G.; Schueller, M.J.; Ferrieri, R.A. Studies Using Mutant Strains of *Azospirillum brasilense* Reveal That Atmospheric Nitrogen Fixation and Auxin Production Are Light Dependent Processes. *Microorganisms* **2023**, *11*, 1727. https://doi.org/10.3390/microorganisms11071727

Academic Editors: Philippe Jeandet, Essaid Ait Barka and Rachid Lahlali

Received: 2 June 2023
Revised: 27 June 2023
Accepted: 29 June 2023
Published: 30 June 2023

Copyright: © 2023 by the authors. Licensee MDPI, Basel, Switzerland. This article is an open access article distributed under the terms and conditions of the Creative Commons Attribution (CC BY) license (https://creativecommons.org/licenses/by/4.0/).

Supplemental Methods

Acetylene Reduction Assay - Calcium carbide (1 g) was placed in a clean and dry 50 mL volume Falcon Tube. The cap to the Falcon Tube was modified to house a Burrell seal port for connecting a 60 mL volume syringe filled with 30 mL DI water and backfilled with 30 mL air for pushing the acetylene out of the Falcon Tube. The cap also held another port for connecting soft silicon rubber tubing connected to the collection bag (Fig. S1). The pouch also had a silicon rubber septum built in for syringe sampling of the gas.

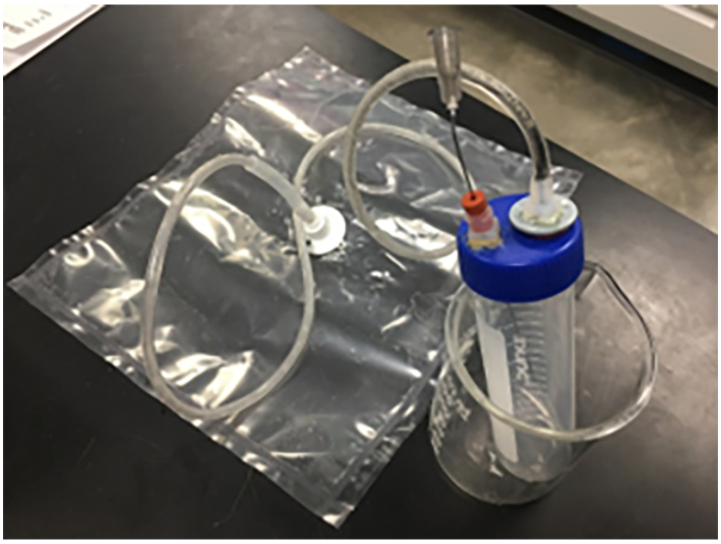

Figure S1. Assembly for preparing batches of acetylene gas for the Acetylene Reduction Assay.

Roots were harvested and sealed inside Mason jars equipped with a sampling port (Fig. S2). Acetylene gas (10 mL volume at standard temperature and pressure (STP)

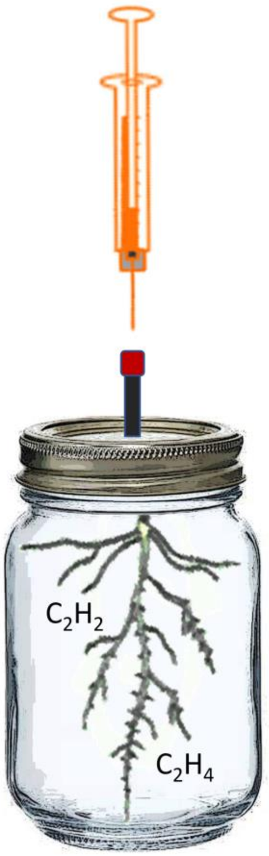

Figure S2. Sample collection jar containing maize roots inoculated with *A. brasilense* bacteria.

was withdrawn from the plastic pouch using a gas tight syringe and injected into the Mason jar through the inlet port. A syringe needle was temporarily placed in the other port to vent the jar of excess pressure. Samples (0.1 mL) were removed at different incubation times for analysis of ethylene production using gas chromatography.

Gas chromatography analysis was performed using a Hewlett Packard 5890A gas chromatograph equipped with a 2-m long, 1-mm inner diameter ShinCarbon ST packed column and flame ionization detector. Conditions included the following: column temperature, 2-min hold at 40 °C then programmed to 250 °C at 10 °C min^{-1}; injector temperature, 250 °C; flame ionization detector temperature, 300 °C. Chromatographic peaks for ethylene were measured using PeakSimple™ chromatography software (v4.95: SRI Instruments, Inc., Torrance, CA, USA) and quantified against ethylene standards.

Drop Plate Assay - Bacterial quantifications were performed concurrently with ARAs to normalize ethylene data to microbial content. Roots were harvested from the growth media and rinsed in DI water. Two separate 3-cm sections of roots were taken from each plant and weighed (approximately 100-300 mg total). The sample was ground with mortar and pestle in 1 mL of 1% saline. Five serial dilutions were conducted: the first with 100 µL of the ground extract into 900 µL of 1% saline and each subsequent dilution being 100 µL of the previous dilution into 900 µL of 1% saline. Each serial dilution was plated in triplicate by 10 µL drops onto agar plates fortified with lactate growth media and incubated at 30 °C for 48-hr before counting. The dilution that contained 3-40 colony forming units (CFU) per 10 µL drop was counted and used to perform calculations of microbial colonization.

Ion Chromatography - The ion chromatography analytical system (Fig. S4) consisted of a Thermo Scientific Dionex AXP Metal-Free HPLC with a Rheodyne metal-free injector and PEEK tubing 1/20 cm inner diameter. The ion chromatography column was a Dionex™ IonPac CS5A column (ThermoFisher Scientific, Inc., Waltham, MA, USA) measuring 4 mm i.d. × 250 mm for the analytical column, and 4 mm i.d. × 40 mm for the CG5A guard column designed to separate a broad range of metal complexes by cation and anion chromatography. The mobile phase consisted of 7 mM pyridine-2,6-dicarboxylic acid, 66 mM potassium sulfate, and 74 mM formic acid pH 4.2 run at a flow rate of 1.2 mL min^{-1}. A post column reagent comprising 0.025 mM luminol and 50 mM sodium bicarbonate pH 10.4 at a flow rate of approximately 0.6 mL min^{-1}. This was mixed downstream with hydrogen peroxide (3% solution) with sodium hypochlorite as a catalyst. Detection was made by

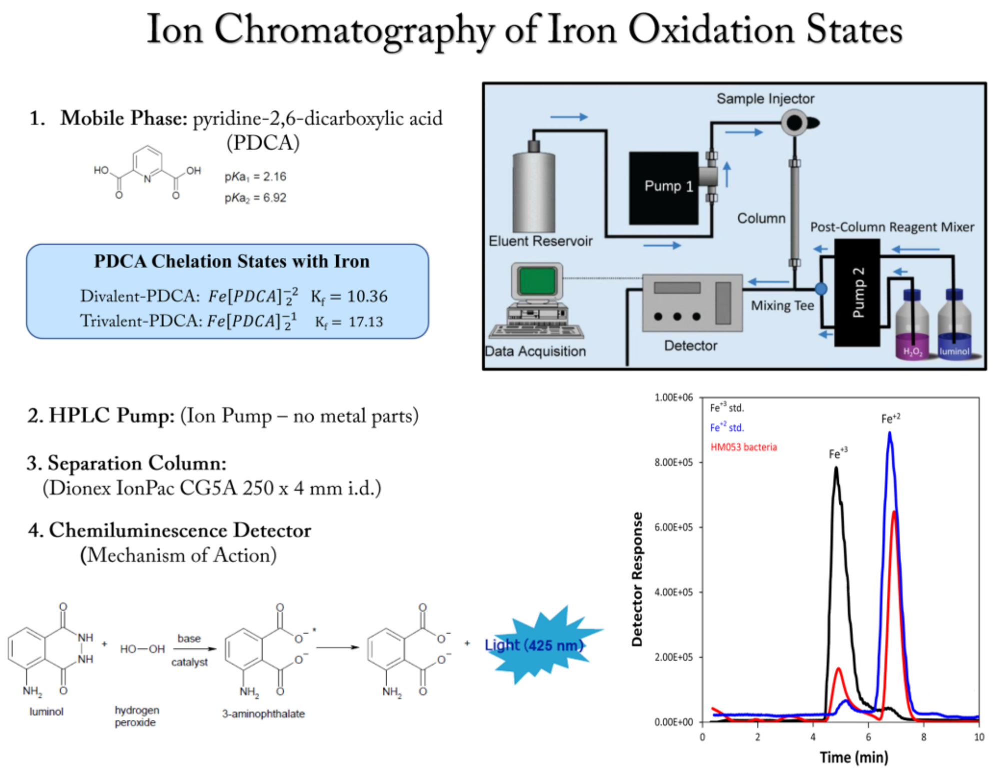

Figure S3. Ion chromatography station used to separate microbial $^{59}Fe^{3+}$ and $^{59}Fe^{2+}$ after radiotracer assimilation.

chemiluminescence using a HERM LB 500 fluorodetector (Berthold GmbH & Co., KG, Germany). Sterile water (HyPure™ WFI Quality Water, HyClone Laboratories, Logan, UT, USA) was used in solvent preparation.

ATP Assay by the Luciferase Method - Bacteria cultures were collected, centrifuged and supernatant separated from the cell pellet. After re-centrifugation 100 µL of the supernatant was mixed with an equal volume of BacTiter-Glo™ Microbial Cell Viability Assay Reagent in a 24-well plate and incubated at room temperature for 5 min. After incubation, luminescence was read using a Typhoon 9000 Imager (imager (Typhoon™ FLA 9000, GE Healthcare, Piscataway, NJ, USA). Bioluminescence for each well was quantified using ImageQuant TL 7.0 software (GE Healthcare, Piscataway, NJ, USA) configured for chemiluminescence mode (Fig. S4). ATP standard solutions were prepared using adenosine 5-triphosphate disodium salt hydrate (Sigma Aldrich, St. Louis, MO, USA) and mixed with equal volumes of the BacTiter-Glo™ Microbial Cell Viability Assay Reagent for calibrating each plate.

Spectrophotometric Auxin Assay - In adapting the colorimetric assay for auxin using the Salkowski reagent (Fig. S5), we applied tryptophan challenges to raise microbial auxin biosynthesis as a test of performance. The Salkowski reagent was prepared by diluting 1 mL of 0.5 M $FeCl_3$ and 24.5 mL of 70% perchloric acid in 24.5 mL distilled H_2O to make a final concentration of 10 mM and 35%, respectively. Sample absorbances were measured

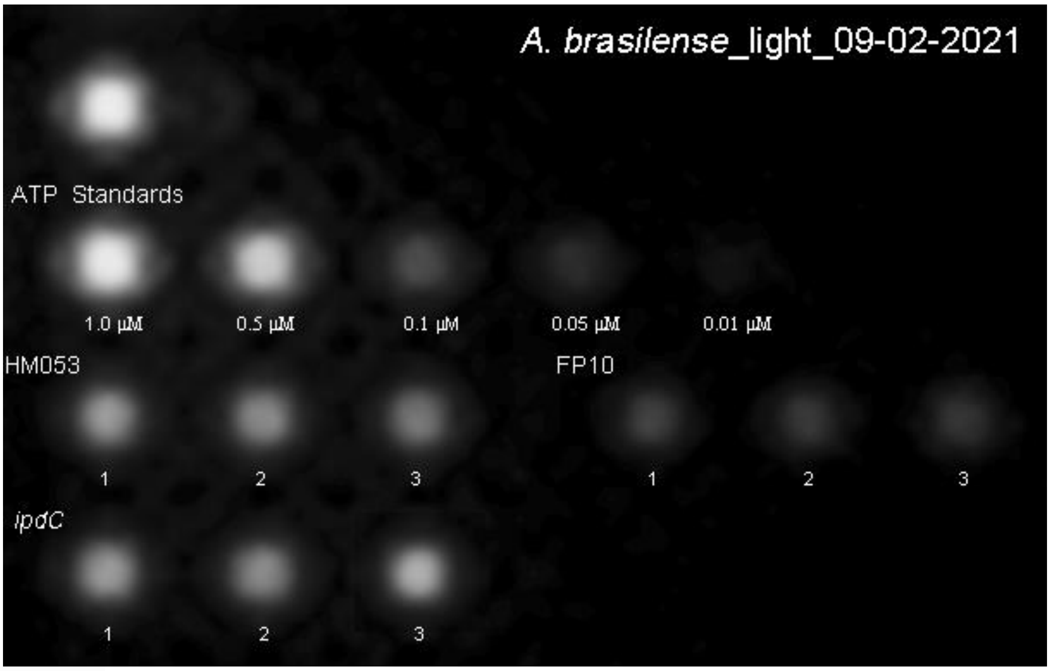

Figure S4. Chemiluminescence spots from the Luciferase Assay using the Typhoon 9000 Imager showing ATP standards and samples done in triplicate for HM053, *ipdC*, and FP10 strains of *A. brasilense* bacteria.

at 536 nm using a spectrophotometer (Evolution 201 UV/VIS, ThermoFisher Scientific Inc., Waltham, MA, USA). For quantitative analysis, serial dilutions from 0 to 50 mg/L of auxin (Sigma Aldrich, St. Louis, MO, USA) were prepared and used as standards.

Cyclotron Production of $^{52}Mn^{2+}$ - Radioactive $^{52}Mn^{2+}$ was produced on the University of Wisconsin PETtrace cyclotron (General Electric Healthcare, Waukesha, WI, USA) by $^{nat}Cr(p,n)^{52}Mn$ using 16 MeV protons. The procedures for radioisotope separation are detailed in the following sentences [34]. Following irradiation, targets were etched with HCl, and the resulting solution was diluted with ethanol. This was done to reach a condition where dissolved Mn^{2+} anionic chloride complexes extract onto strong anion-exchange resin while hydrated Cr^{3+} passes through. Upon switching to a fully aqueous 6 M HCl solution $^{52}Mn^{2+}$ was released from the resin bed. The entire procedure of trap-and-release, alternating from ethanolic to aqueous mixtures was repeated three times on three separate small AG-1 × 8 columns. To render the purified dose of $^{52}Mn^{2+}$ in 0.5 mL of dilute aqueous HCl for shipment.

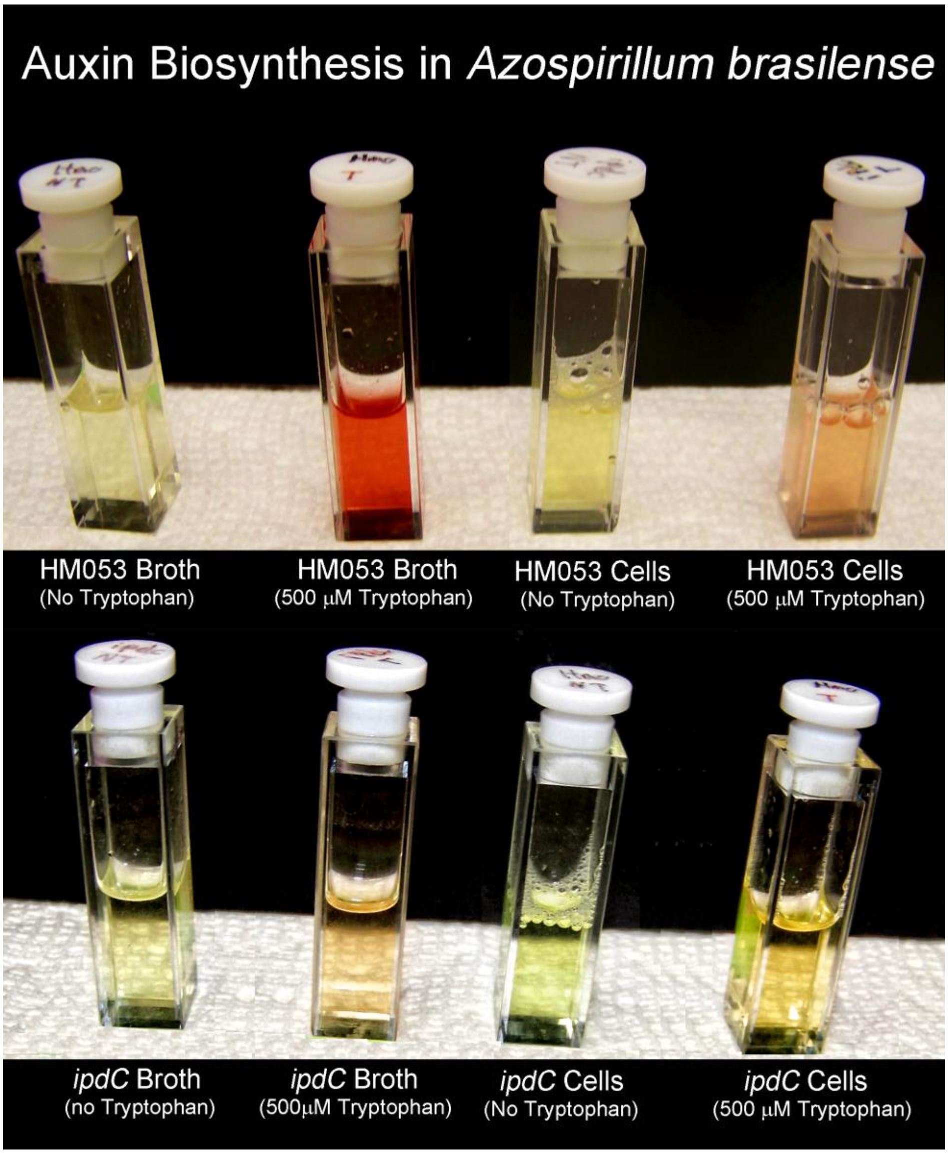

Figure S5. Optical cuvettes showing color differences between HM053, a high auxin producing strain, and *ipdC*, an auxin deficient strain of *A. brasilense* bacteria. When developing the method, we applied tryptophan as a challenge to elevate microbial auxin levels.
